# CXCR4^hi^ effector neutrophils in sickle cell anemia: potential role for elevated circulating serotonin (5-HT) in CXCR4^hi^ neutrophil polarization

**DOI:** 10.1038/s41598-020-71078-8

**Published:** 2020-08-31

**Authors:** Flavia Garcia, Rafaela Mendonça, Lediana I. Miguel, Venina M. Dominical, Sara T. O. Saad, Fernando F. Costa, Nicola Conran

**Affiliations:** 1 Hematology Center, University of Campinas – UNICAMP, Rua Carlos Chagas 480, Cidade Universitária, Campinas, SP 13083-878 Brazil; 2 Flow Cytometry Core Facility, National Heart, Lung, and Blood Institute, NIH, Bethesda, MD USA

**Keywords:** Mechanisms of disease, Haematological diseases

## Abstract

Leukocyte recruitment and heterocellular aggregate formation drive the inflammatory vaso-occlusive processes associated with sickle cell anemia (SCA). We characterized neutrophils in a population of patients with SCA and investigated whether platelet-derived molecules can induce phenotypic alterations in this cell type. Imaging flow cytometry analysis demonstrated that the frequency of circulating CXCR4^hi^ neutrophils was significantly higher in steady-state SCA individuals than in healthy control individuals and that these cells presented increased CD11b activation and toll-like receptor-4 expression. SCA neutrophils display increased neutrophil-platelet aggregation, and CXCR4^hi^ neutrophils demonstrated augmented neutrophil-platelet aggregate frequency with a higher mean number of platelets adhered per neutrophil. Importantly, incubation of neutrophils with platelets significantly elevated their CXCR4 expression, while SCA plasma was found to induce CXCR4^hi^ neutrophil polarization significantly more than control plasma. SCA individuals had significantly increased plasma levels of serotonin (5-HT), and serotonin molecule and SCA plasma induced neutrophil CXCR4 expression in a serotonin-receptor-dependent manner. Thus, the augmented CXCR4^hi^ neutrophil population may contribute to mechanisms that promote vaso-occlusion in SCA; furthermore, circulating serotonin, derived from platelet activation, may play a role in the polarization of neutrophils, suggesting that serotonin-receptor antagonists or serotonin reuptake inhibitors could represent therapeutic approaches to reduce neutrophil activation in SCA.

## Introduction

Despite being a monogenic disease, sickle cell anemia (SCA) incurs major damage to various organs and systems. The replacement of an adenine nitrogen base with a thymine nitrogen base in the hemoglobin β-globin gene results in the substitution of the apolar amino acid, valine (Val), in place of the polar amino acid, glutamic acid (Glu), at position six of the polypeptide chain, resulting in the production of the anomalous hemoglobin, hemoglobin S (HbS). HbS, when deoxygenated, polymerizes into elongated fibers that compromise the flexibility of erythrocytes, rendering them sickle-shaped, very susceptible to lysis and with different adhesive and physical properties^1,2^.

In addition to alterations in their erythrocytes, individuals with SCA display evidence of leukocyte, endothelial and platelet activation, as a result of processes of ischemia/reperfusion and intravascular hemolysis^3–5^. These activated cells, in turn, direct the progression of inflammatory processes and participate in the vaso-occlusive events that characterize the disease. Among the leukocytes involved in the pathophysiology of SCA, neutrophils are particularly relevant, as these are the most abundant leukocytes in the circulation and, when activated, are recruited to vascular walls, in turn, forming aggregates with erythrocytes and/or platelets through interactions involving the integrin α_M_β_2_ (Mac-1; CD11b/CD18)^6,7^. Neutrophils are granular leukocytes that contain multilobulated nuclei and were, until recently, considered to belong to a homogenous, short-lived, effector cell population with a restricted function, targeting extracellular microorganisms, mainly by means of phagocytosis. More recently, however, the longevity of neutrophils has been questioned and these cells are now recognized as being extremely versatile and heterogeneous^8^. Indeed, certain neutrophil phenotypes have been associated with characteristic inflammatory contexts^9^, with reports of a prevalence of PDL-1^high^ (Programmed Death Ligand-1) neutrophils in HIV-positive patients^10^ and CD15^+^CD16^low^ neutrophils in cancer patients^11^, both acting as negative regulators of T lymphocyte proliferation. Additionally, aged neutrophils (defined as CD62L^lo^ CXCR4^hi^ neutrophils) have been described to present a highly reactive effector phenotype that may provide a “first response” during innate immune mechanisms and in patients and mice with SCA^12,13^.

On the other hand, platelets also appear to play a very important role in SCA, not only due to their role in the hypercoagulable state and their contribution to heterotypic cellular aggregates^3,4,14,15^, but also due to the fact that they secrete soluble factors that act on neighboring cells. In SCA, platelets are generally believed to circulate in an activated state, given the expression of P-selectin and phosphatidylserine on their surface^15,16^; importantly, when activated, platelets can release more than 300 types of bioactive substances, including serotonin, or 5-hydroxytryptamine (5-HT)^17^. Serotonin is not produced by platelets, rather this amine is synthesized by enterocromaffin cells and neurons of the enteric nerve plexus and Central Nervous System. Serotonin produced by enterocromaffin cells, and released into the blood, is taken up by platelets, which then store it in dense granules and secrete it back into the bloodstream after exposure to certain agonists^17–20^. Recently, platelet serotonin has been shown to be able to recruit neutrophils to the endothelium^21^, increase vascular permeability^22^ and, surprisingly, induce phenotypic change in monocytes and neutrophils, favoring the activation of a pro-inflammatory profile^23,24^. In the present study, we characterize of circulating CXCR4^hi^ neutrophils in a population of individuals with SCA, identifying these CXCR4^hi^ neutrophils as displaying some effector functions, and demonstrate that this population is subject to regulation by serotonin, released as a consequence of platelet activation in the disease.

## Results

### Patients with SCA have a higher frequency of circulating CXCR4^hi^ neutrophils than healthy control individuals

**Figure 1:**
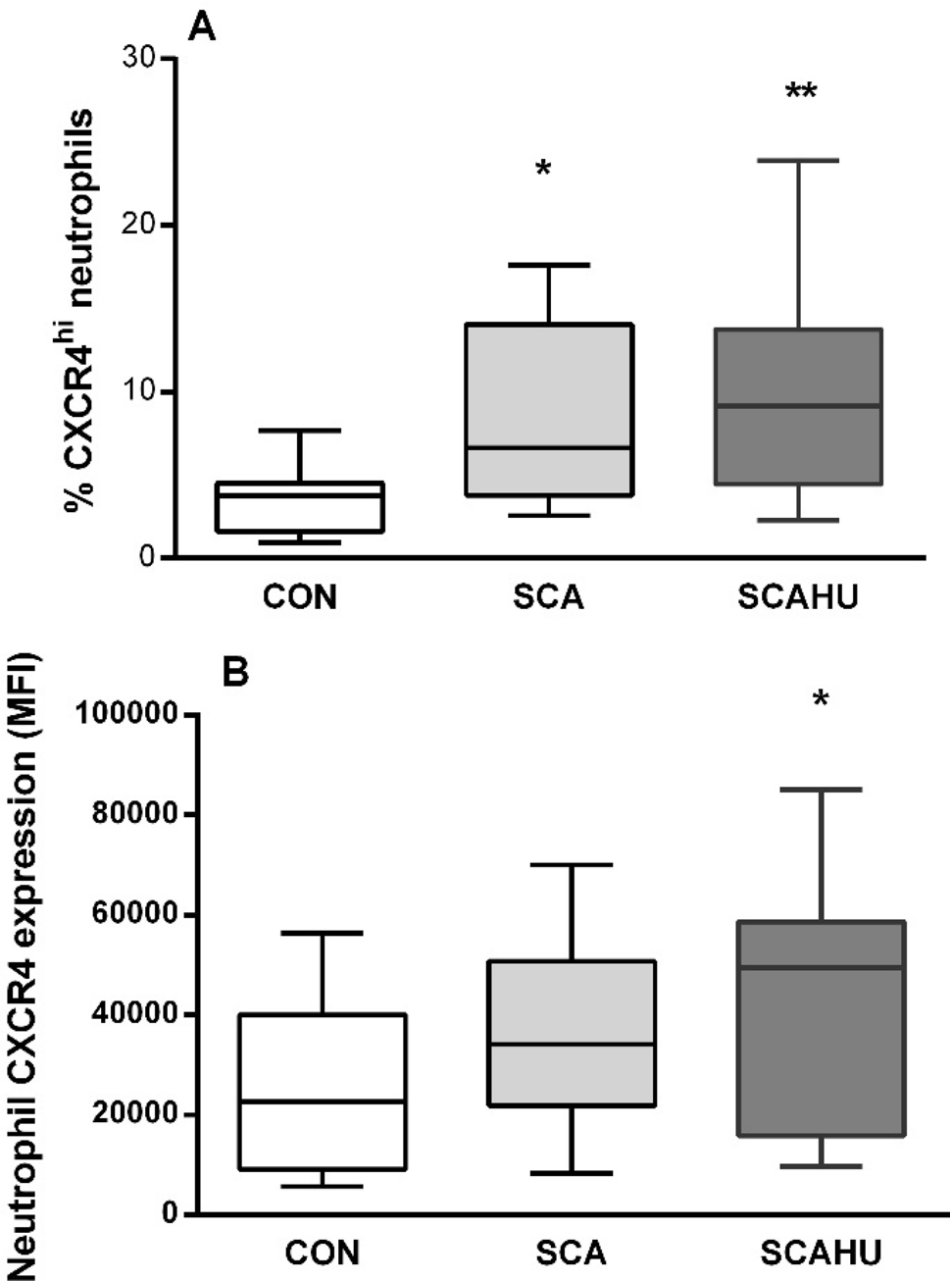
The CXCR4^hi^ neutrophil subset is increased in SCA. (**A**) Percentage of the total of neutrophils that presented a high surface expression of CXCR4 (CXCR4^hi^ neutrophils) in the peripheral blood of healthy control (CON, n = 16) individuals and steady-state SCA patients off (SCA, n = 9) and on hydroxyurea (SCAHU, n = 12) therapy, determined by flow cytometry. (**B**) Density of expression (Geo mean fluorescence intensity) of CXCR4 on the surface of neutrophils. Kruskall–Wallis test, Dunn's multiple comparison test. *P ≤ 0.05 and **P ≤ 0.01, compared to CON.

To confirm neutrophil heterogeneity in SCA, we initially investigated whether our population of patients with the disease present neutrophils of the CXCR4^hi^ phenotype. Flow cytometry analysis showed that the percentage of the total of neutrophils that presented a high surface expression of CXCR4 (CXCR4^hi^ neutrophils) was significantly higher in patients with SCA, when compared to healthy control individuals (Fig. 1A). Interestingly, the frequency of neutrophils demonstrating this phenotype did not differ significantly between those patients on and off therapy with hydroxyurea (HU), the drug most commonly used to treat the disease^25^ (Fig. 1A). In association with the increased percentage of neutrophils presenting high surface CXCR4 expression in SCA patients, the increase in the relative expression of CXCR4 per cell, as demonstrated by mean fluorescence intensity (MFI), was statistically significant on neutrophils from SCA individuals on HU therapy (Fig. 1B).

### Phenotypic profile of CXCR4^hi^ neutrophils

**Figure 2:**
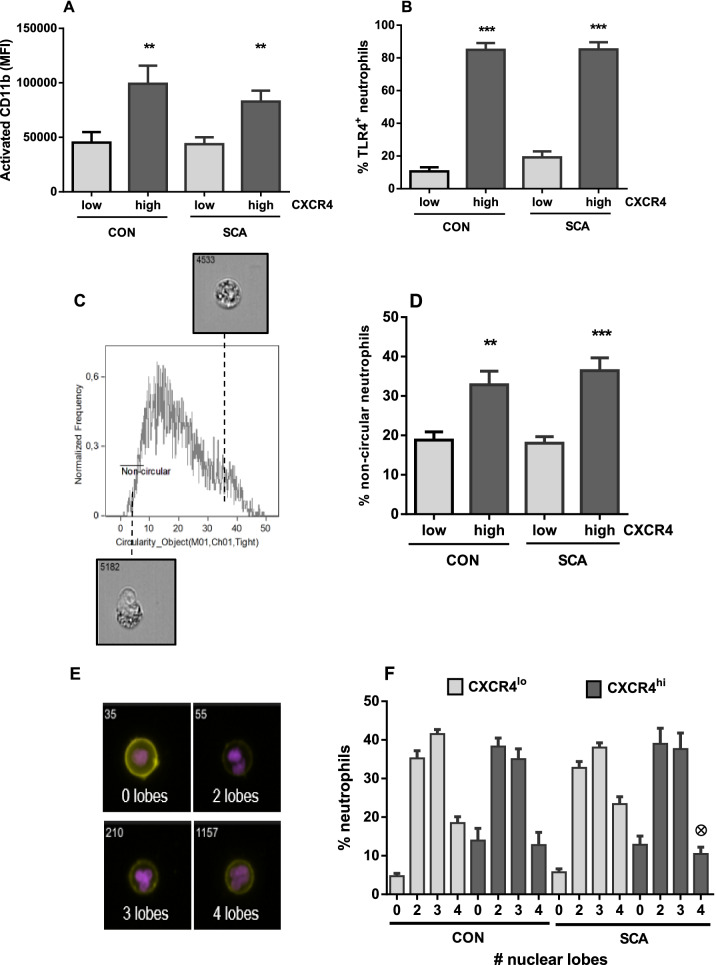
Phenotypic profile of CXCR4^hi^ neutrophils. Density of CD11b activation (**A**) and frequency of TLR-4 expression (**B**) on CXCR4^low/neg^ (low) and CXCR4^hi^ (high) neutrophils of CON (n = 10, n = 7, respectively) and SCA individuals (n = 21, n = 17, respectively). (**C**) Representative histogram depicting circularity distribution and representative images of non-circular (left) and circular (right) cells, as determined by imaging flow cytometry, used for calculating (**D**) the frequency of non-circular cells in the CXCR4^low/neg^ and CXCR4^hi^ neutrophil populations of CON (n = 16) and SCA individuals (n = 29). (**E**) Representative images, obtained by imaging flow cytometry, of cells containing one, two, three or four nuclear lobes, and (**F**) frequency of cells containing one, two, three or four nuclear lobes in the CXCR4^low/neg^ and CXCR4^hi^ neutrophil populations of CON (n = 11) and SCA individuals (n = 20). ^*^P ≤ 0.05, ^**^P ≤ 0.01 and ^***^P ≤ 0.001, compared to CXCR4^low/neg^ neutrophils; ⊕ *P* ≤ 0.05, compared to CXCR4^low/neg^ neutrophils (4 lobes); (ANOVA, Dunn's multiple comparison test).

Having established the high frequency of CXCR4^hi^ neutrophils in SCA individuals, we next characterized their potential to act as effector cells in SCA, evaluating the activation of the Mac-1 beta integrin chain (CD11b) on the surface of these cells, as well as their expression of Toll-Like Receptor 4 (TLR4). CXCR4^hi^ neutrophils, in both SCA patients and control subjects, presented a higher density of CD11b in its activated conformation, compared to the density of activated CD11b on other neutrophils (CXCR4^low/neg^ neutrophils; Fig. 2A). Approximately 90% of CXCR4^hi^ neutrophils were also positive for TLR4 expression in both control subjects and SCA patients, in contrast to less than 20% of TLR4-positive cells among the CXCR4^low/neg^ neutrophils (Fig. 2B).

In addition to the membrane expression profile, we also evaluated neutrophil morphology using the IDEAS software tools. Neutrophils are circular cells, when at rest; however, certain neutrophil activities, such as phagocytosis, release of neutrophil extracellular traps (NETs), interaction with other cells and cell migration can incur morphological changes. The frequency of non-circular cells was significantly higher in the CXCR4^hi^ neutrophils, in both patients with SCA and in control individuals (Fig. 2C,D).

To further evaluate the phenotypic profile of CXCR4^hi^ neutrophils, we investigated the degree of maturity of these cells using a frequency analysis of cells containing zero, two, three or four nuclear lobes. Interestingly, there was a reduction in the frequency of cells containing 4 lobes in CXCR4^hi^ neutrophils, compared to CXCR4^low/neg^ neutrophils in SCA samples (Fig. 2E,F). Shedding of surface CD62L is also a hallmark of neutrophil aging; however, the CD62L expression density on the CXCR4^hi^ neutrophils studied herein was not lower than that of CXCR4^low/neg^ neutrophils in either the control subjects or the SCA subjects (Supplementary Figure 1). CD62L expression density was, though, lower on SCA CXCR4^hi^ neutrophils than on control subject CXCR4^hi^ neutrophils (Supplementary Figure 1). These data indicate that the circulating CXCR4^hi^ neutrophil population, under analysis, are not necessarily aged neutrophils, but that they present major phenotypic alterations.

Additionally, the phenotypic comparison of CXCR4^hi^ neutrophils from patients on and off HU therapy demonstrated that this neutrophil subset did not differ significantly in those patients on HU, compared to those patients that were not taking HU (Supplementary Figure 2A–F), with regard to CD11b activation, TLR4 expression, non-circularity and number of lobes.

### Participation of CXCR4^hi^ neutrophils in the formation of aggregates with platelets

**Figure 3:**
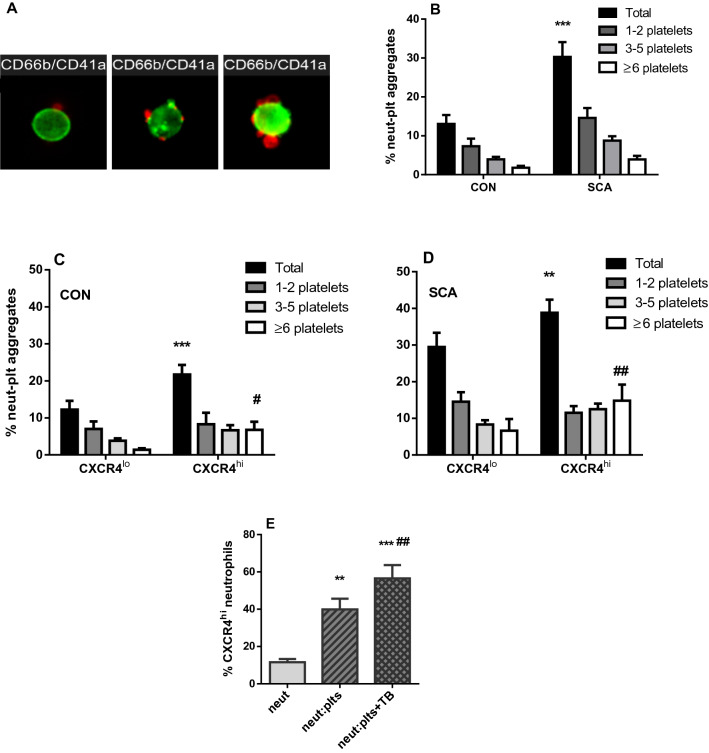
Participation of CXCR4^hi^ neutrophils in the formation of aggregates with platelets. (**A**) Representative images, obtained by imaging flow cytometry, depict anti-CD66b-FITC (green, neutrophils) and anti-CD41a-PerCp e-Fluor 710 staining (red, platelets), with varying numbers of platelets aggregated to neutrophils. (**B**) Percentages of neutrophils aggregated with platelets; total percentage of neutrophils involved in aggregates and percentages of neutrophils aggregated with 1–2, 3–5 and ≥ 6 platelets per neutrophil in CON (n = 7) and SCA (SCA n = 12) subjects. ***P ≤ 0.001, compared to CON; Two-way ANOVA, Sidak’s post test. (**C**,**D**) Total frequency of neutrophils involved in aggregates and neutrophil aggregates formed by 1–2, 3–5 and ≥ 6 platelets within the CXCR4^low/neg^ neutrophil group and within the CXCR4^hi^ neutrophil group in neutrophils from (**C**) healthy (CON; n = 7) and (**D**) SCA (n = 12) individuals. **P ≤ 0.01; ***P ≤ 0.001, compared to total aggregates in CXCR4^low/neg^; ^#^*P* ≤ 0.05, ^##^*P* ≤ 0.01, compared to CXCR4^low/neg^ (≥ 6 plts) (Two-way ANOVA, Sidak’s post test). (**E**) Soluble platelet-derived mediators induce neutrophil polarization into the CXCR4^hi^ phenotype. Neutrophils were isolated from healthy individuals (n = 5) before incubating without/with platelets (separated by 0.4 μm pore size transwell inserts) in the presence/absence of 0.1 U/mL thrombin (TB; 37 °C, 5% CO_2_) for 2 h. Figure depicts percentage of neutrophils that were CXCR4^hi^ following incubation of neutrophils, determined by flow cytometry. **P ≤ 0.01; ***P ≤ 0.001, compared to neutrophils alone; ^##^*P* ≤ 0.01, compared to neutrophils incubated with platelets. (ANOVA, Holm-Sidak’s multiple comparison test).

The quantification of the neutrophil-platelet aggregates present in the peripheral blood of healthy individuals and patients with SCA was made using imaging cytometry, as described in “Methods” section. Following thirty minutes of static incubation of peripheral blood (4 °C, in the dark), about 13.0 ± 2.3% and 30.3 ± 3.8% (*P* < 0.001) of neutrophils displayed platelets adhered on their surface, for control subjects and patients with SCA, respectively (Fig. 3A,B). Hydroxyurea therapy was not associated with any significant alteration in the profile of platelet-neutrophil aggregations in SCA individuals (Supplementary Figure 2G).

We then examined the participation of CXCR4^hi^ neutrophils of CON and SCA individuals in the formation of heterotypic aggregates with platelets. CXCR4^hi^ neutrophils from both control individuals and patients with SCA presented higher frequencies of aggregate formation and higher numbers of aggregates formed with 6 or more platelets, than CXCR4^low/neg^ neutrophils (Fig. 3C,D).

### Soluble platelet-derived mediators induce neutrophil polarization into the CXCR4^hi^ phenotype

Having confirmed CXCR4^hi^ neutrophils as potential effector cells in SCA pathophysiology, and their participation in the formation of neutrophil-platelet aggregates, we explored whether soluble platelet-derived factors may induce neutrophil polarization to the CXCR4^hi^ phenotype. Neutrophils were incubated (37 °C) in the upper sections of transwell chambers and separated, by inserts, from platelets in the lower wells of chambers, in the presence or absence of thrombin (0.1 U/ml). After 2 h of incubation, there was a significant increase in the percentage of CXCR4^hi^ neutrophils when cells were incubated in the presence of platelets, and this increase was even greater in the presence of thrombin (Fig. 3E). These results indicate that platelets may induce neutrophil CXCR4 expression and that this effect is mediated by soluble factors derived from platelets and not by direct cell–cell contact.

### Platelet-derived serotonin is a plasma soluble factor that induces CXCR4^hi^ phenotype polarization in neutrophils

**Figure 4:**
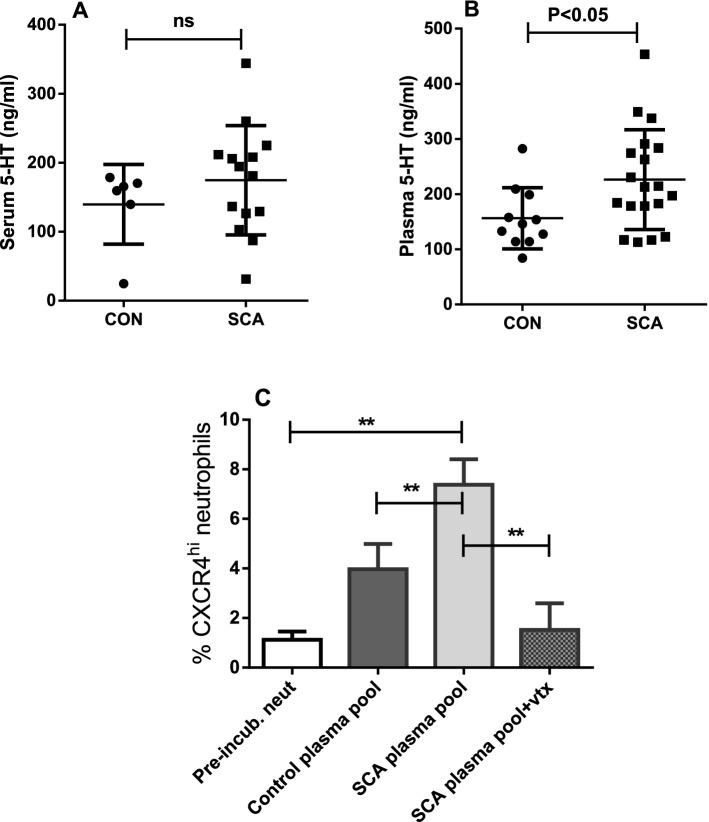
Platelet serotonin induces the CXCR4^hi^ phenotype in neutrophils. (**A**) Serum serotonin (5-HT) levels in samples from the CON group (n = 6) and samples from the SCA group (n = 14), determined by ELISA. (**B**) Plasma levels of serotonin in samples from the CON group (n = 11) and in samples from the SCA group (n = 19), determined by ELISA (Mann–Whitney test). (**C**) Quantification of the percentage of CXCR4^hi^ cells, following incubation of neutrophils from healthy individuals (n = 3) with 10% (v/v) of a pool of plasma from control subjects (pooled n = 12) or from patients with SCA (pooled total n = 16; SCA = 8; SCAHU = 8), and in the presence of the pooled SCA plasma containing 60 nM vortioxetine (vtx, non-selective inhibitor of 5-HT receptors). **P ≤ 0.01 (ANOVA, Holm-Sidak’s multiple comparison test).

As thrombin is a strong platelet agonist, inducing the release of its granular contents into the extracellular medium, we evaluated whether the serum and plasma levels of one of the substances stored in the dense platelet granules, serotonin (5-HT), may be altered in patients with SCA, in relation to those of control subjects. Notably, there was no difference in serum amine levels between the groups studied (Fig. 4A); however, higher plasma serotonin levels were detected in SCA patients, compared to the control group (Fig. 4B). HU therapy did not have any significant effect on serum or plasma serotonin levels. Plasma 5-HT was 203.8 ± 16.3 ng/ml and 246.8 ± 36.4 ng/ml in SCA [n = 9] and SCAHU [n = 10] patients, respectively (*P* > 0.05).

Additionally, we evaluated whether the plasma of control subjects or the plasma of patients with SCA could influence neutrophil polarization to a CXCR4^hi^ phenotype. Indeed, following incubation of neutrophils from control individuals with 10% (v/v) plasma pooled from SCA individuals, the percentage of CXCR4^hi^ neutrophils was significantly elevated, compared to the percentage of CXCR4^hi^ neutrophils observed in the absence of plasma or when incubated with plasma pooled from control individuals (Fig. 4C). Furthermore, a non-selective serotonin receptor inhibitor, vortioxetine (vtx, 60 nM), abolished the increase in the percentage of CXCR4^hi^ neutrophils stimulated by the pool of plasma from patients with SCA (Fig. 4C).

### Serotonin induces neutrophil polarization to the CXCR4^hi^ phenotype, in vitro

**Figure 5:**
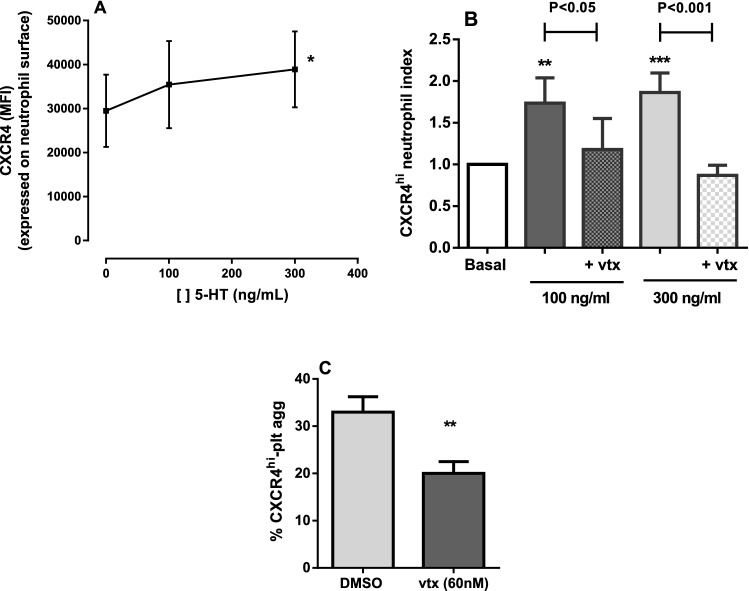
In vitro effects of serotonin (5-HT) and vortioxetine (vtx) on neutrophil polarization and neutrophil-platelet aggregate formation. Surface CXCR4 expression density (**A**) and CXCR4^hi^ neutrophil index (**B**) after neutrophils from healthy individuals were incubated (37 °C, 5% CO_2_) for 4 h with different concentrations of purified serotonin in the presence, or not, of 60 nM vtx, a non-selective serotonin receptor inhibitor. CXCR4^hi^ neutrophil index corresponds to the ratio between the percentage of CXCR4^hi^ neutrophils in samples with serotonin and the percentage of CXCR4^hi^ neutrophils in samples without serotonin.*P ≤ 0.05, **P ≤ 0.01 and ***P ≤ 0.001, compared to 0 ng/mL; n ≥ 6 (ANOVA, Dunn’s multiple comparisons test). (**C**) Whole blood samples from control subjects were incubated with either 60 nM vtx or DMSO (2 h) and, after incubation, the percentage of platelet-containing neutrophils was evaluated (n = 6). **P ≤ 0.01; paired *t*-test.

The in vitro effect of purified serotonin on neutrophil polarization was evaluated by incubating neutrophils, isolated from healthy control individuals, with different concentrations of serotonin for 4 h. At the concentration of 300 ng/ml, serotonin significantly increased CXCR4 expression density (as demonstrated by Mean Fluorescence Intensity of CXCR4 staining) on the surface of neutrophils from healthy individuals (Fig. 5A), while at concentrations of 100 ng/ml and 300 ng/ml, serotonin significantly increased the relative percentage of CXCR4^hi^ neutrophils, compared to neutrophils incubated in medium alone (Fig. 5B). Importantly, the addition of the non-selective 5HT receptor inhibitor, vtx (60 nM), abolished the effect of serotonin on CXCR4 polarization (Fig. 5B), without affecting cell viability (as confirmed by Annexin V/ 7-amino-actinomycin [7ADD] staining; data not shown). Furthermore, when whole blood, obtained from control subjects, was incubated for 2 h at 37 °C in the presence of vtx (60 nM), the percentage of CXCR4^hi^ neutrophils involved in platelet aggregate formation was significantly decreased (Fig. 5C).

## Discussion

Leukocytes, especially neutrophils, play a central role in initiating and driving the inflammatory and vaso-occlusive processes that cause many of the clinical complications of SCA^5^. Although it is possible that different subpopulations of neutrophils may originate from the segregation of a common lineage still in the bone marrow, recent observations indicate that neutrophil heterogeneity is the result of their response to peripheral stimulation^9–11^. In SCA, the extracellular medium contains numerous stimulating substances that are derived from lysed erythrocytes, activated leukocytes and platelets, including reactive oxygen species, proinflammatory cytokines, enzymes, and soluble versions of adhesion molecules^1,3^. Many of these substances have paracrine actions and may favor the predominance of certain phenotypic leukocyte profiles.

In the present study, consistent with previous observations^12^, SCA patients demonstrated a higher frequency of CXCR4^hi^ neutrophils, indicating the existence of a pathophysiological environment conducive to neutrophil polarization to this phenotype. CXCR4^hi^ neutrophils may have great relevance to the pathophysiology of SCA, since these cells exhibit a high density of surface molecules known to be involved in neutrophil priming and function^6,26^, including TLR4, which plays an important role in mediating cellular innate immune responses^26^. The increased activation of the Mac-1 integrin observed on these CXCR4^hi^ neutrophils also confers important properties to these cells^6^, facilitating cell recruitment to the vascular wall and adhesive interactions^27^. Indeed, we found that CXCR4^hi^ neutrophils captured significantly more platelets on their cell surface than CXCR4^low/neg^ neutrophils, indicating that these cells probably make an important contribution to the increased heterotypic aggregate formation observed in SCA and that is associated with vaso-occlusion and pathophysiology in the disease^7,28^. The neutrophils characterized, herein, as CXCR4^hi^ neutrophils (according to criteria described in “Methods” section) displayed some important effector functions, but could not all be denominated as aged neutrophils^29,30^ since, in general, the CXCR4^hi^ neutrophil population did not display CD62L shedding, or increased nuclear lobe formation, compared to CXCR4^low/neg^ neutrophils. Thus, the CXCR4^hi^ neutrophil subset, described herein, may or may not contain a population of aged neutrophils, as defined by other authors^12,13^.

Interestingly, both SCA patients on and off HU therapy presented a higher frequency of CXCR4^hi^ neutrophils than control subjects; furthermore, the phenotypic profile of the CXCR4^hi^ neutrophils identified did not differ between those patients that were taking HU, compared to those that were not. It is difficult to determine whether the lack of an association between HU therapy and modulation of CXCR4^hi^ neutrophil frequency in SCA indicates a lack of effect of this therapeutic molecule^31^ on the factors that drive neutrophil polarization, or whether this observation may be due to HU indication bias^32^. Presently, in centers that heavily rely on HU as a therapeutic approach for SCA, patients that are not on HU therapy are usually patients that have a less severe clinical course^33^.

Platelet-neutrophil aggregates are formed by multiple ligands, but evidence suggests that they are principally formed by interactions between platelet surface P-selectin and neutrophil surface P-selectin glycoprotein ligand 1 (PSGL-1) and between platelet glycoprotein Ib (GPIb) and the neutrophil Mac-1 integrin^34^. We found the Mac-1 subunit, CD11b, to be significantly more activated on CXCR4^hi^ neutrophils in both control and SCA subjects, suggesting that this ligand may play a role in the elevated propensity of these cells to form these heterocellular aggregates. However, a role for P-selectin-PSGL-1 interactions in the CXCR4hi neutrophil aggregates observed should not be ruled out. Given the increased interaction that CXCR4^hi^ neutrophils display with platelets, we determined whether platelets and/or factors released by these cells may influence neutrophil phenotype. Importantly, we found that factors released from platelets, and especially from activated platelets, can significantly induce the polarization of neutrophils to the CXCR4^hi^ phenotype. It should be noted that, in addition to our observation of a likely influence of platelet activation on neutrophil phenotype, it is also probable that neutrophil polarization to the CXCR4^hi^ phenotype may also influence platelet activation. Activated neutrophils are known to release several soluble platelet-activating mediators^34^, and, while we did not evaluate NET generation by the CXCR4^hi^ neutrophils, stimulation of neutrophil CXCR4 expression with a low dose of lipopolysaccharide has been associated with the release of NETs, which in turn have documented platelet activating and thromboinflammatory effects ^35,36^.

In association with these findings, plasma from individuals with SCA was also found to significantly induce CXCR4 expression on healthy control neutrophils. Interestingly, serotonin, one of the substances released during activated platelet degranulation, has been previously shown to control the expression of CXCR4 on lymphocytes^37^ by modulating the response of these cells to CXCL12. We found that serotonin was significantly elevated in the plasma of patients with SCA. No differences in serum serotonin levels were observed between the studied groups, probably due to the fact that serum contains not only plasma serotonin but also the serotonin that is stored in platelets^38^. Platelets release serotonin after stimulation with immunological complexes, and it is possible that, during episodes of high platelet activation, as may occur in SCA^39^, much of the serotonin stored in platelets is released into the extracellular medium and that platelets with empty dense granules are maintained in the circulation^40^.

The incubation of neutrophils with purified serotonin, at physiologically relevant concentrations, induced polarization of neutrophils to the CXCR4^hi^ phenotype. Importantly, inhibition of serotonin receptors was able to abolish the effect of both SCA plasma and of purified serotonin on neutrophil polarization and also reduced the ability of these cells to form heterocellular interactions with platelets, indicating a central role for serotonin-mediated signaling in driving the phenotype and function of neutrophils. Long-term serotonin reuptake inhibition (SRI), which reportedly protects patients with depression from cardiovascular events, has been shown to deplete platelet serotonin stores in mice, while plasma serotonin levels correlated with neutrophil CD11b expression in patients hospitalized with acute coronary syndrome, indicating that inhibition of serotonin uptake may decrease neutrophil activation^24^.

As such, our data confirm a polarization of neutrophils to the CXCR4^hi^ phenotype in SCA and demonstrate that this polarization confers properties to these neutrophils that are extremely relevant to the pathophysiology of SCA. Importantly, the release of serotonin from activated platelets appears to play an important role in this polarization, suggesting the use of serotonin receptor antagonists as a therapeutic approach for diminishing neutrophil activation in SCA.

## Methods

### Individuals participating in the study

Patients homozygous for hemoglobin S (HbSS; total of 50 patients) were recruited from the clinical outpatient clinic of the Hematology and Hemotherapy Center of UNICAMP (Supplementary Table 1). All patients were in steady state and had not been hospitalized due to vaso-occlusive episodes or received blood transfusion during the previous 3 months. Some patients were on hydroxyurea (HU; total of 32 patients) therapy (SCAHU; 15–30 mg HU/kg/day for at least 2 months). The control group (CON) was formed by blood donors enrolled at the same health care unit; controls were age- and sex-matched where possible. Both patient samples and control subjects were collected in the morning so that the results would not be hampered by chronobiological variations. This study was approved by the Ethics Committee of the University of Campinas-UNICAMP (Protocol #: 48964115.1.0000.5404), and all protocols were carried out in accordance with the Declaration of Helsinki, relevant guidelines and regulations; all study participants provided their informed and signed consent.

### Isolation of granulocytes

Granulocytes were isolated from peripheral blood for quantification of CXCR4^hi^ neutrophils and evaluation of neutrophil-platelet clusters. Blood samples (4 ml) were collected in EDTA and centrifuged over a Histopaque gradient (1.077 g/ml ≤ 1.119 g/ml, Sigma Aldrich), according to previously described methodology^41^. Leukocytes located in the density layer > 1.077 g/ml ≤ 1.119 g/ml were collected for quantification of CXCR4^hi^ neutrophils, while for the quantification of neutrophil-platelet clusters, the leukocytes/platelets were obtained from both the > 1.077 g/ml ≤ 1.119 g/ml and ≤ 1.077 g/ml layers and were mixed. In both cases, cells were washed with 0.1 M PBS and the concentration of the cell suspension was adjusted to 2 × 10^7^ cells/ml in RPMI medium.

### Peripheral blood counts

Peripheral blood counts of patients were obtained by counting cells in an automatic counter (Beckman Coulter AcT Diff Hematology Analyzer, Brea, California, USA).

### Phenotypic analysis of neutrophils using imaging cytometry

After separation of granulocytes, as described above, cells were incubated for 30 min with the fluorescent antibodies specified below, in the dark at 4 °C. Data from approximately 10,000 cells, with areas ranging from 100 to 250 μm^2^, were acquired on an imaging cytometer (Amnis Imagestream Mark II, Luminex Corp, TX, USA) using the 405 nm, 488 nm and 642 nm lasers and image amplification set to 40 × or 60 ×—and analyzed through IDEAS software (Luminex).

### Definition of CXCR4^hi^ neutrophils and evaluation of CD11b activation and TLR4 expression

The CXCR4^+^ neutrophil population was defined by positive events for Brilliant Violet 421TM anti-human CD184 (CXCR4) antibody (clone 12G5; Biolegend, CA, USA) within the "CD66b morphology" mask, used for neutrophil identification. The "CD66b morphology" mask included all fluorescent pixels of labeling for the FITC anti-CD66b monoclonal antibody or PE anti-CD66b monoclonal antibody (clone G10F5 and clone G10F5, respectively; eBioscience, Thermo Fischer, MA, USA) and was employed to prevent possible errors in the distinction between CXCR4 expressed on neutrophils and the same molecule expressed on platelets. Finally, within the CXCR4^+^ neutrophil population, CXCR4^hi^ neutrophils were defined as CXCR4^+^ events with fluorescence values above the geometric fluorescence mean of CXCR4. PerCP-eFluor 710 anti-CD11b monoclonal antibody (activation epitope-specific, clone CBRM1/5; eBioscience), PE-anti human CD62L (clone DREG-56, BD Biosciences, CA, USA) and Alexa Fluor 488 anti-CD284 (TLR4) monoclonal antibody (eBioscience, clone HTA 125) were used to analyze CD11b activation, CD62L expression and TLR4 expression, respectively.

### Evaluation of heterotypic neutrophil-platelet (Neut-Plt) clusters

Samples were labeled with anti-CD66b FITC, anti-CD41a PerCp e-Fluor 710 (eBioscience, clone HIP8) and anti-CXCR4 BV421 antibodies. The analysis of platelets that were adhered to neutrophils was performed using an adaptation of the method described by Hui et al.^42^. Briefly, the "internalization" analysis tool of the IDEAS software was applied, so that the "internalization" of the CD41a PerCp e-F710 (platelet) was measured inside the “neutrophil membrane mask”, which was defined by subtracting the eroded 5-pixel CD66b mask from the CD66b Morphology mask. The frequencies of different numbers of platelets per neutrophil in these aggregates were obtained by the additional *Wizard Spot Count* application, which gives the number of bright images inside an object, in this case, the number of platelets (bright images of CD41a) in the aggregates.

### Lobe count

Cells were labeled with DRAQ5 Fluorescent Probe (Thermo Fisher) to stain the cell nucleus (Fig. 2E). A mask was created including all DRAQ5 pixels, and denominated Nucleus Morphology, and the Lobe Count tool of the IDEAS software was applied to this mask^43^. The Lobe Count tool measures the number of lobes in the nucleus, based on the greatest tendency of the image (Nucleus Morphology) to have a single axis of elongation (Symmetry 2 or 2 lobes), three axes (Symmetry 3 or 3 lobes), four axes (Symmetry 4 or 4 lobes) or no axis at all (Symmetry 1).

### Quantification of serum and plasma serotonin

Serum was obtained by collection of peripheral blood in tubes without anticoagulant; blood coagulation occurred within approximately 2 h, after which the sample was centrifuged for collection of the serum (1,000*g*, 20 min, room temperature). Plasma was obtained by collection of blood in EDTA tubes, followed by centrifugation (1,000*g*, 15 min, room temperature). Quantification of serum or plasma serotonin was performed using an ELISA for the quantification of human serotonin (Abbexa, Cambridge, UK).

### Platelet separation

Peripheral blood was collected in tubes with the anticoagulant sodium citrate (ACD) and centrifuged at room temperature, 200 g, 20 min. The supernatant was collected, centrifuged at 800 g (RT, 12 min) before resuspending in Krebs buffer (1.26 M NaCl, 25 mM KCl, 250 mM NaHCO_3_, 12 mM NaH_2_PO_4_, 12 mM MgCl_2_, 25 mM CaCl_2_).

### Co-incubation of neutrophils and platelets physically separated by transwell inserts

Transwell inserts containing filters and pores of 30 mm and 0.4 μm in diameter, respectively (Merck, Darmstadt, Germany), were used to separate platelet neutrophils in 6-well culture plates. Co-incubation was performed in the presence/absence of 0.1U/mL thrombin at 37 °C in a humidified 5% CO_2_ atmosphere for 2 h.

### Incubation of neutrophils with serotonin

Granulocytes isolated from control subjects were incubated (37 °C, 5% CO_2_) for 4 h in the presence of different concentrations of serotonin (hydrochloride, R&D Systems). As neutrophils exhibit circadian variations in the pool of circulating cells expressing CXCR4^hi^^44^, and these assays were performed at different times of the day, the data regarding percentages of CXCR4^hi^ neutrophils were normalized by calculating the ratio between the values of samples containing serotonin and the values of samples without serotonin in each test (CXCR4^hi^ neutrophil index).

### Statistical analysis

Values are expressed as means ± standard error of mean (SEM). Data were confirmed as parametric, or not, and comparisons were performed using ANOVA with multiple comparisons post-tests, as appropriate and as specified. Differences among groups were considered to be significant at *P* ≤ 0.05.

## Supplementary information

Supplementary information.

## Data Availability

All data derived from this study are included in this article. Datasets can be provided from the authors upon request.
