# Supplementary material for: CXCR4hi effector neutrophils in sickle cell anemia: potential role for elevated circulating serotonin (5-HT) in CXCR4hi neutrophil polarization
Source: Sci Rep. 2020 Aug 31;10:14262. doi: 10.1038/s41598-020-71078-8 (PMC7459317; doi:10.1038/s41598-020-71078-8)
Supplement: Supplementary file 1 — Supplementary information. [file 41598_2020_71078_MOESM1_ESM.pdf]

CXCR4^{hi} effector neutrophils in sickle cell anemia: Potential role for elevated circulating serotonin (5-HT) in CXCR4^{hi} neutrophil polarization

Flavia Garcia, Rafaela Mendonça, Lediana I. Miguel, Venina M. Dominical, Sara T.O. Saad, Fernando F. Costa and Nicola Conran

Supplementary Material

Supplementary Table 1. Characteristics and hematological parameters of SCA patients included in the study

**Parameter**	
Age (years)	38.56±1.36 (38, 18, 75)
Male/Female	22/28
HU therapy (-/+)	18/32
WBC (10^3/µL)	7.97±0.44 (7.53, 2.82, 18.64)
Neut (10^3/µL)	4.21±0.36 (3.79, 1.05, 15.49)
RBC (10^6/µL)	2.53±0.09 (2.53, 1.35, 4.9)
Hb (g/dL)	8.71±0.24 (8.5, 3.8, 13. 2)
HCT (%)	26.21±0.71 (26.10, 12.40, 36.80)
PLT (10^3/uL)	375.3±18.54 (361, 148, 726)
HbF	14.42±1.34 (13, 3.6, 33.8)

The values in parentheses correspond to the median, minimum and maximum.
WBC: White Blood Cell count, Neut: Neutrophil count, RBC: Red Blood Cell count, Hb: Hemoglobin, HCT: Hematocrit, PLT: Platelet count, HbF: Fetal Hemoglobin.

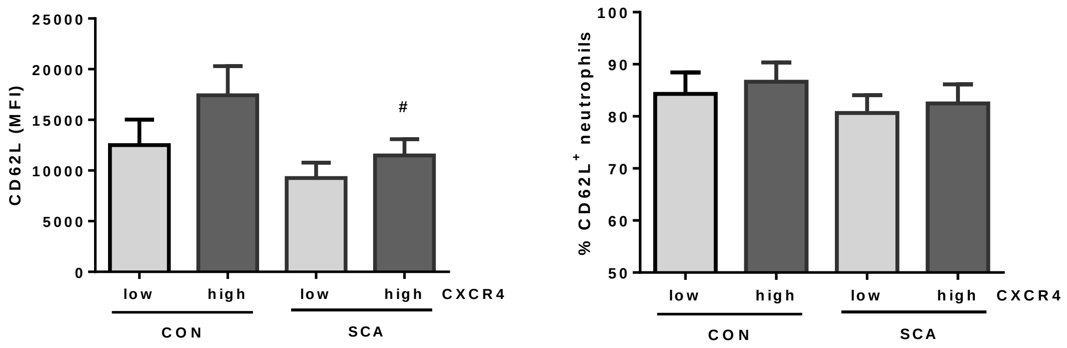

Supplementary Figure 1. Density of expression (A) and percentage of CD62L on the surface of CXCR4$^{lo/neg}$ and CXCR4^{hi} neutrophils of CON (n=14) and SCA individuals (total n=22; SCA=10; SCAHU = 15), as determined by flow cytometry. #P≤0.05, compared to CXCR4^{hi} neutrophils in CON subjects (ANOVA, Holm-Sidak's multiple comparison test). No significant differences were observed between the SCA and SCAHU neutrophil populations.

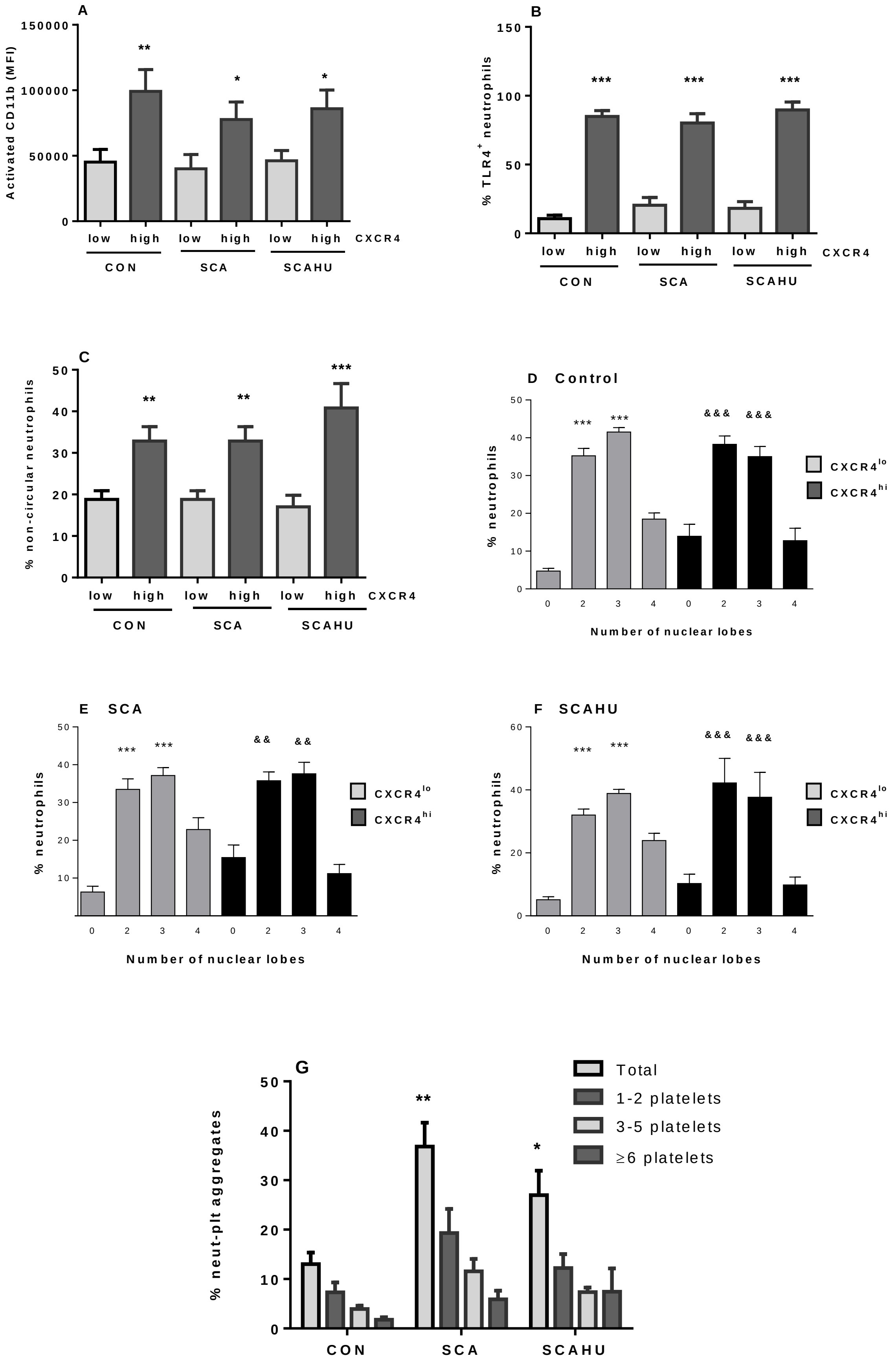

Supplementary Figure 2.

Supplementary Figure 2 Legend: (A-F) Phenotypic profile of CXCR4^{hi} neutrophils from healthy control subjects (CON), SCA patients (SCA) and SCA patients on HU therapy (SCAHU). Density of CD11b activation (A), frequency of TLR-4 expression (B) and non-circular cell frequency (C) for CXCR4^{hi} neutrophils and CXCR4$^{low/neg}$ neutrophils from CON (n≥7), SCA (n≥8) and SCAHU (n≥9) individuals, determined by flow cytometry. Frequency of cells containing one, two, three or four nuclear lobes in the CXCR4^{hi} neutrophils and CXCR4$^{low/neg}$ (CXCR4^{lo}) neutrophils of CON (D, n=11), SCA (E, n=10) and SCAHU (F, n=10) individuals. *P≤0.05, **P≤0.01 and ***P≤0.001, compared to CXCR4$^{low/neg}$ neutrophils; &&, P≤0.01, &&&, P≤0.001, compared to CXCR4$^{low/neg}$ neutrophils. (ANOVA, Sidak's multiple comparison test). (G) Frequency of neutrophils forming aggregates with 1-2, 3-5 and ≥6 platelets for neutrophils from CON (n=7), SCA (n=4) and SCAHU (n=8) individuals. *P≤0.05, ** P≤0.01; compared to total aggregates in CON subjects (Two-way ANOVA, Sidak's post test).
